# The Gendered Impact of the COVID-19 Pandemic on Employment in Argentina: The Mediating Role of the Public vs. Private Sectors

**DOI:** 10.3390/socsci13020123

**Published:** 2024-02-19

**Authors:** Yasmin A. Mertehikian, Emilio A. Parrado

**Affiliations:** Department of Sociology, University of Pennsylvania, Philadelphia, PA 19104-6299, USA

**Keywords:** COVID-19, Argentina, gender, work, public sector, private sector

## Abstract

This study examines the COVID-19 pandemic’s immediate and long-term impact on Argentina’s labor market with a focus on gender disparities and the mediating role of the public vs. private sectors. Using household survey data, we assess men and women’s employment trends before, during, and after the pandemic. Our findings reveal gender-specific recovery patterns that interact with the employment sector. The most prominent short-term effect of the pandemic was a dramatic increase in inactivity for both men and women. However, men recovered their level of labor force participation sooner than women, and one of the mechanisms behind this disparity was sector employment. While men predominantly benefitted from quicker reintegration in both the formal and informal private sectors, women leaned toward the public sector for stability during and after the pandemic. The heightened feminization of public sector employment is a further indication that the sector is critical for sustaining women’s employment and promoting gender equity in the labor market.

## 1. Introduction

The COVID-19 pandemic prompted governments around the world to implement quarantine and social distancing measures in an effort to stop its spread. However, these measures were associated with reduced economic activity and, consequently, led to a severe economic crisis on a global scale. Millions faced unemployment, curtailed working hours, or were forcibly ousted from the workforce (International Labour Organization (ILO) 2021; Richter 2021). Latin America was among the regions most detrimentally impacted by the pandemic’s economic fallout. The economic contraction witnessed in the region surpassed that of both advanced and emerging economies (Arreaza et al. 2021). Within Latin America, the most precipitous declines in economic activity were recorded in Panama (−18%), Peru (−11%), and Argentina (−9.9%) (Arreaza et al. 2021). These drops in economic activity were larger than those observed in developed countries like the United States, where the GDP fell by 9% during the first months of the pandemic (Center on Budget and Policy Priorities 2024).

The pandemic’s effects have not been homogeneous across social and demographic groups. Numerous studies have revealed that women suffered more in terms of employment, hours of work, income, and labor force participation compared to men (e.g., Collins et al. 2021; Hipp and Bünning 2021; Mertehikian and Gonalons-Pons 2022; Reichelt et al. 2021). Women also faced an uptick in child caregiving burdens (e.g., Dunatchik et al. 2021; Farré et al. 2020; Manzo and Minello 2020; Zamarro and Prados 2021). In Latin America, the pandemic’s adverse impact on women’s employment was compounded by their overrepresentation in informal employment (e.g., Ameijeiras et al. 2021; Arreaza et al. 2021; Batthyány and Sánchez 2020; Bergallo et al. 2021; Ernst and López Mourelo 2020; Gutiérrez et al. 2020). Specifically, in Argentina, while both men and women initially faced increased inactivity due to the pandemic, men rebounded faster a year later, whereas women, especially the younger and less educated, remained disproportionately affected (Mertehikian and Parrado 2024).

How did the COVID-19 pandemic affect different employment sectors in Argentina? How did these changes vary across genders? Our study aims to answer these questions by examining the roles of the public and private sectors in Argentina’s labor market during and after the pandemic, with a focus on gender differences. While substantial empirical evidence has shown the pandemic’s gendered impact on employment, the extent of sectoral shifts within the labor market remains underexplored. Few studies differentiate the roles of the public and private sectors during and after the pandemic. Ameijeiras et al. (2021) documented that women in Argentina moved from informal roles to the public sector more than men. However, they did not assess short- and longer-term effects, as well as the dynamics of employment transitions.

Differentiating between the roles of the public and private sectors as employers is pivotal in the context of Latin American labor markets. Historically, public employment has been leveraged by governments in the region as a tool to mitigate the adverse impact of frequent economic crises. The public sector is a significant employment source in Latin America, especially for women. Its share in salaried employment has increased in recent decades, particularly since the 2000s, and especially in South America (Economic Commission for Latin America and the Caribbean (ECLAC) et al. 2013; Gasparini et al. 2015). The public sector has played a crucial role in creating jobs during recessive periods, acting as a buffer against soaring unemployment and inactivity rates that could have been even more pronounced (Fernández and González 2020; Marshall 1990). Latin American governments have historically achieved this either through the direct creation of new public jobs—temporary or permanent—or by retaining existing positions (Farné 2016; Marshall 1990).

Argentina presents an intriguing case for two main reasons. Firstly, the country, akin to Venezuela, has seen a rise in public employment over recent decades, accounting for nearly 20% of total jobs during the 2010s—significantly above the regional average of about 12% (Diéguez and Gasparin 2016; Fernández and González 2020; Gasparini et al. 2015; Organization for Economic Cooperation and Development (OECD) 2016, 2020). Secondly, there is a discernible gendered trend in employment across Latin America and distinctly in Argentina. Women have become predominantly employed in the public sector, particularly in roles deemed traditionally “female”, such as public health and education, while men dominate the private sector (Dávila 2008; Gasparini et al. 2015; Organization for Economic Cooperation and Development (OECD) 2016, 2020). Indeed, over 50% of women in Argentina are employed within the public sector (Organization for Economic Cooperation and Development (OECD) 2016, 2020).

We argue that a more complete account of the gendered impact of the pandemic on employment needs to differentiate between the roles of the public and private sectors as well as short- and longer-term effects. Our results show that while the pandemic initially affected men and women similarly, men recovered their level of labor force participation sooner than women. One of the mechanisms behind the disparity was sector employment. Informal private employment declined similarly for both genders immediately because of the pandemic. However, while informal sector employment recovered for men, that was not the case for women. Not only did women face greater difficulties in returning to informal employment, but they actually continued to transition out of informality and into inactivity well into the pandemic. Similar gender differences were evident for formal employment in the private sector. By contrast, only public formal employment showed no gender differences in the degree of protection afforded to workers. More interesting, the expansion of formal sector employment to respond primarily to the health needs of the Argentine population further attracted women into public formal employment. Therefore, while the private sector reactivated men’s employment, the public sector has been central in reactivating women’s employment.

## 2. Context

### 2.1. The Political Response to the COVID-19 Pandemic in Argentina

As with many nations globally after the COVID-19 outbreak, Argentina enacted measures to control and stop virus spread and mitigate labor market consequences. In March 2020, it imposed Preventive and Mandatory Social Isolation (PMSI) (Castagna et al. 2021). PMSI entailed a complete shutdown of non-essential activities, followed by a gradual, activity-based reopening (Mera et al. 2021). The most severe restrictions occurred from March to October 2020, representing the pandemic’s peak impact on Argentina’s labor market. Subsequently, most economic activities resumed and mobility restrictions were lifted, returning the country to a certain pre-pandemic normality by early 2021. The educational system was an exception to this pattern. Argentina, like other countries in the region, closed its schools from 16 March 2020 until around mid-February 2021, although there were variations in school reopenings throughout 2021 according to the epidemiological situation of the country and of each region (Arnold et al. 2021; Matovich and Bucciarelli 2021; Rodríguez 2021; Ruiz and Cornaglia 2023; Schwal 2022; United Nations International Children’s Emergency Fund (UNICEF) 2021). Only by the end of September 2021 did classes resume fully in person (Ruiz and Cornaglia 2023). This placed Argentina eighth in the world and second in South America in terms of the number of weeks that schools were closed (United Nations Educational, Scientific and Cultural Organization (UNESCO) 2021, cited in Ruiz and Cornaglia 2023, p. 2). School closings had a direct impact on women with children, as they faced increased challenges in managing both work and family responsibilities (Arza 2020; Passerino and Trupa 2020; Wigdor and Bonavitta 2021).

Argentina went through the COVID-19 pandemic amid the government of Alberto Fernández, who was part of *Kirchnerism*. *Kirchnerism* is a political movement that emerged in Argentina after the 2001 crisis and a left-wing faction within the Peronist party in the country. The Kirchner administrations ruled the country from 2003 to 2015 and from 2019 to 2023. These administrations showed clear statist leaning—that is, they were governments in which the state had a predominant role in the economy. This was reflected both in greater market regulations as well as the consequent expansion of public employment (Calvo and Murillo 2012). Such ideology explains the policy responses adopted to mitigate the effects of the pandemic, particularly in economic terms. Indeed, public health measures coincided with policies aimed at protecting employment and vulnerable workers. Drawing from Mera et al. (2021) and Ernst and López Mourelo (2020), government initiatives can be separated by whether they are oriented toward the protection of formal or informal workers. Policies oriented toward the informal sector focused on replacing lost labor income with government subsidies for unemployed or low-income workers. Initially, the government expanded existing programs like the Universal Child Allowance (AUH, due to its acronym in Spanish^1^) and the Universal Pregnancy Allowance (AUE, in Spanish) while introducing emergency programs. The Emergency Family Income (IFE, for its acronym in Spanish), created in April 2020, provided payments equal to 60% of the minimum wage to eligible individuals based on their employment status or income. These programs continued but were adjusted as the labor market evolved.

Formal sector measures were oriented toward protecting jobs during the pandemic. From March 2020 to December 2021, the government prohibited dismissals and suspensions. Financial assistance compensated employers for salaries of all formal employees in companies with up to 100 workers as well as healthcare workers. The Emergency Assistance Program for Work and Production (ATP, due to its acronym in Spanish) supported companies in salary payments. This program ended in late 2020, replaced by the Productive Recovery Program (REPRO, in Spanish) for companies with declining revenues. The government also reduced employer contributions to social security for severely affected companies and deferred consumption tax (VAT) payments for small and medium-sized enterprises. As economic recovery advanced, these policies were gradually cut down.

### 2.2. Female Labor Force Participation and Public Employment in Argentina

Gender differences in the short- and longer-term impacts of the pandemic and their interactions with employment were also grounded in the particular characteristics of women’s work in Argentina, and Latin America more generally. Over the past decades, Argentina has witnessed growing participation of women in the labor force. The pattern mimics the trends for Latin America. For the region as a whole, women’s labor force participation increased from 20% in the 1960s to 65% in the pre-pandemic period (Frisancho et al. 2023; Marchionni et al. 2019). Argentina, in turn, experienced a nearly 22 percent point increase (from 26.5% to 48.1%) between 1970 and 2017 (Manzano 2015; Ministerio de Trabajo, Empleo y Seguridad Social 2017). These changes were highly significant for reducing gender disparities in economic resources. The expansion of female employment reduced the gender gap in labor force participation from 54.1 to 22.4 percentage points during the same period (Manzano 2015; Observatorio de la Violencia contra las Mujeres 2018). By 2019, just before the COVID-19 pandemic, women’s activity rate in Argentina was 49.2%, while men’s was 70.2% (Rulli 2020).

This rise in female labor force participation coincided with the growth of public sector employment in both the region and the country. Public employment has historically served as a buffer against unemployment during crises and as a cushion against insufficient private sector job creation (Farné 2016; Fernández and González 2020; Marshall 1990). The role of public employment has been especially critical in Latin America, where economic crises are frequent. During the period spanning from 2012 to 2014, 12% of the employed population reported working in the public sector in the region (Gasparini et al. 2015; Organization for Economic Cooperation and Development (OECD) 2016). This percentage remained stable through 2018 (Organization for Economic Cooperation and Development (OECD) 2020), despite a considerable increase in the number of public employees since 2000—which in the case of Argentina was nearly 70% (Diéguez and Gasparin 2016). Argentina stands out in the region concerning public employment, as public employment’s share of the labor market reached almost 20% in 2014 (Diéguez and Gasparin 2016; Organization for Economic Cooperation and Development (OECD) 2016), and the significant public sector representation remained until 2018 (Organization for Economic Cooperation and Development (OECD) 2020). Furthermore, Argentina is one of four countries in the region that showed an increase in the share of public employment in total employment from 2011 to 2018 (from 16.2% to 17.2%) (Organization for Economic Cooperation and Development (OECD) 2020). This increase took place particularly in the context of *Kirchnerist* governments, for which public sector employment was not only part of their core ideology in economic terms but also constituted a way of reaching their constituencies (Calvo and Murillo 2012).

The growth of the public sector though is highly gendered. Like the general trend in the region (Organization for Economic Cooperation and Development (OECD) 2020) and in more developed countries (Gornick and Jacobs 1998; Organization for Economic Cooperation and Development (OECD) 2016, 2020; Schmidt 1993, cited in Gornick and Jacobs 1998, p. 688), women in Argentina are overrepresented among public sector workers (Organization for Economic Cooperation and Development (OECD) 2016, 2020; Secretaría de Gestión y Empleo Público de la Nación 2022). This is because the public sector tends to employ more women than men, especially in occupations traditionally considered “female”, such as public education and health services. It also offers better conditions than the private sector for women balancing work and family obligations (Dávila 2008; Organization for Economic Cooperation and Development (OECD) 2016, 2020). The latter became more evident in the context of the COVID-19 pandemic when public sector employment—except in the case of the health sector—allowed for remote working, providing more job stability and security for women.

Currently, over half of public sector employees in Latin America are women (Organization for Economic Cooperation and Development (OECD) 2020). This contrasts with the private sector, in which men’s labor force participation significantly exceeds that of women (Dávila 2008; Gasparini et al. 2015). As Gasparini et al. (2015) noted, women’s greater participation in public sector employment occurs in a context in which men are experiencing a decline in this sector. This decrease, driven by growing female labor force participation in the region, has also occurred in the private sector, albeit to a lesser extent. While the proportion of men in public employment fell by 10 percentage points between 1992 and 2012, the private sector saw only a 5-point decline (Gasparini et al. 2015, p. 757). However, the importance of the public sector as a source of employment for women becomes more evident when analyzing its influence on women’s employment rates. In 2011, 17% of employed women in Latin America were salaried employees in the public sector, compared to 10.3% of men; in Argentina, this was even more pronounced, with 22.5% of women being salaried employees in the public sector for the same year (Economic Commission for Latin America and the Caribbean (ECLAC) et al. 2013, p. 47).

Together, the trends in gender differences in public and private sector employment as well as the policy responses to the pandemic underscore clear interactions between gender, the employment sector, and the varying duration of the pandemic’s impact on employment.

## 3. Data and Methods

We investigated these interactions using cross-sectional and panel data from nine waves of the Argentine household survey (*Encuesta Permanente de Hogares de Argentina*, EPH). The data spanned from the fourth quarter of 2019 to the fourth quarter of 2021. These quarters encompassed pre-pandemic conditions (Q4 2019 and Q1 2020), the immediate aftermath of the pandemic outbreak (Q2, Q3, and Q4 of 2020 as the short term), and the subsequent economic recovery period one and a half years post-outbreak (all of 2021 as a longer-term perspective). Data from the National Institute of Statistics and Census (INDEC, for its acronym in Spanish) indeed showed this trend: while Argentina was already under an economic recession during the first quarter of 2020, the second quarter of that year represented a disastrous contraction with a decline in the GPD of almost 15.8% compared to the previous quarter (INDEC 2021a). It was only by the first quarter of 2021 that the national GDP saw a positive net growth in comparison to the year before (of almost 2.9%) (INDEC 2021b). The transition from the third quarter of 2021 to the fourth quarter of that year, moreover, picked up those still left behind by the economic recovery. The EPH is a household survey offering quarterly data, based on a stratified probabilistic sample from 32 urban centers in Argentina with 100,000 or more inhabitants. The EPH follows a 2-2-2 scheme, wherein households within an area are surveyed for two consecutive quarters, then omitted for the next two quarters, and finally surveyed again for another two consecutive quarters. This design facilitates both cross-sectional analysis and tracking individual labor transitions longitudinally across quarters.

We limited our sample to individuals aged 18 to 64 at the time of the survey to capture the primary working age population. The dependent variable categorized respondents into six mutually exclusive employment statuses: out of the labor force, employed in the informal private sector, employed in the public formal sector, employed in the private formal sector, unemployed, and self-employed. Informal employment was defined, in line with Argentina’s National Institute of Statistics and Census, as salaried workers without pension contributions.^2^ To distinguish whether a respondent was employed in the public or private sector, we relied on their response to the survey question about the nature (state-owned or private) of the institution/business where they worked most weekly hours.

Gender was a central dimension framing this analysis, and the results are presented separately for men and women. For the cross-sectional assessment, we conducted *t*-tests for each employment status to test gender differences in the percentages of men and women in each occupational status across quarters, taking the first quarter of 2020 as the reference period. The longitudinal analysis further narrowed the sample to the panel of respondents with data before, during, and/or after the pandemic and explored individual employment transitions for both genders.

## 4. Results

### 4.1. Cross-Sectional Results

The gender distribution within employment statuses in Argentina showed a clear pattern, as shown in Table A2 in the Appendix A: while men consistently represented a larger percentage of the labor force within the informal and formal private sectors, women were overrepresented in public sector employment over the period of analysis. This distribution, in turn, correlated with how each sector responded during and after the pandemic. Table 1 shows the employment status percentages of men and women from Q4 2019 to Q4 2021. In the pre-pandemic period, men and women’s inactivity rates remained steady. However, by Q2 2020, inactivity rose sharply to 27.2% for men and 48.0% for women. By 2021, these rates returned to pre-pandemic figures. Informal employment decreased with the pandemic’s onset, recovering by the beginning of 2021 among men and toward the end of that year among women. Formal public employment initially remained stable, but from Q1 2021, an increase was observed, much more among women (14.1%) than among men (12.2%). Formal private sector employment initially decreased slightly, with men recovering faster by the beginning of 2021. Unemployment remained stable until late 2021, decreasing to around 5% for both genders. Lastly, self-employment initially declined but bounced back by Q4 2020.

Table 2 displays the quarter-by-quarter differences in the percentages of each employment status during and after the pandemic compared to the first quarter of 2020—that is, the quarter preceding the onset of COVID-19—for both men and women. The table also displays the results of *t*-tests to ascertain whether these differences are statistically significant. The findings from Table 2 illustrate both a general trend in the pace of recovery in labor market position, as well as an overall description of employment sectors accounting for these shifts during the pandemic. Before the COVID-19 pandemic in Q4 2019 and Q1 2020, employment status percentages for both genders remained stable in Argentina. However, by Q2 2020, the pandemic significantly increased inactivity levels by around 11 percentage points for both genders. As inactivity rose, employment in the vulnerable informal and self-employment sectors dropped: men saw decreases of 7.8 and 4.7 points, respectively, while women experienced drops of 6.7 and 3.8 points. Interestingly, employment in the formal sector remained stable during Q2 2020. Comparing Q1 and Q3 of 2020, gender differences in labor market recovery emerged. Men exhibited a quicker recovery, with an inactivity rise of 5.3 points from Q1, while women saw a 6.7-point increase. Men also rebounded faster in informal and self-employment. By Q3, men’s informal employment was down by 4.8 points from Q1, compared to women’s 5.1-point decrease. In self-employment, men gained 1.1 points, but women dropped 0.7 points. Both genders experienced declines in the formal private sector: −1.4 points for men and −1.0 for women. However, public employment remained stable, indicating its buffering role against the pandemic’s initial impact, as supported by regional literature. By Q4 2020, these trends deepened, with men’s recovery outpacing women’s, particularly in informal employment.

Table 2 also contrasts post-pandemic inter-quarterly employment status variations against Q1 2020 for both genders. By Q1 2021, distinct gender disparities emerged. Men’s inactivity rate declined by 1.4 points, suggesting faster reintegration into the Argentine labor market compared to women.^3^ By Q1 2021, men also regained their pre-pandemic formal private employment levels, while women lagged by 1.1 points. Similar gender differences were evident for informal employment: while men recovered pre-pandemic informal private employment levels during Q1 2021, women lagged behind by 2.7 points. This quarter also indicated a growing feminization trend in formal public employment: while both genders saw increases, women’s rise was 0.6 points more than men’s (1.3 vs. 0.7 points, respectively). This gap expanded to 1.8 points in Q2 2021. By Q4 2021, both genders’ inactivity levels reverted to below pre-pandemic levels. However, distinct gender patterns emerged: the percentage of men in the formal private sector grew by 2.5 points from Q1 2020, while women’s rose by only 0.8 points. By the end of 2021, women’s public employment surpassed pre-pandemic levels more than men’s, with gains of 1.3 vs. 0.1 points, underscoring the feminization trend in Argentina’s public sector.

### 4.2. Panel Data Results

In this section, we present the panel data results for individuals with information on employment status before, during, and/or after the pandemic to analyze specific transitions between employment categories. Table 3 reports changes in employment transitions for men and women during two periods: (a) during the immediate impact of the COVID-19 pandemic, and (b) after the reopening of economic activities in Argentina. For analytical purposes, we focused on three quarters: Q2 2020 (during the pandemic), Q1 2021, and Q4 2021 (both post-pandemic). Tables A3 and A4 in the Appendix A include the results for the nine quarters in our sample. The following set of analyses provides a detailed description of individual employment transitions over the analyzed period, allowing for a more comprehensive understanding of labor processes in the country.

The panel data results presented in Table 3 highlight the mechanisms explaining the trends described with the cross-sectional data regarding the immediate and longer-term impacts of the pandemic. Firstly, focusing on the short-term impact of the pandemic, and considering employment transitions from before the pandemic (Q1 2020) to during the pandemic (Q2 2020), the results revealed a substantial increase in inactivity among both men and women. This rise in the percentage of inactive individuals can be attributed to the transition of men and women previously employed in the informal sector and self-employed, as well as those who were unemployed before the pandemic. Indeed, 27.6% of men and 41.5% of women in informal employment became inactive during the early months of the pandemic. At the same time, 29.8% and 39.8% of men who were previously self-employed and unemployed shifted to inactivity during Q2 2020, while for women these percentages were 35.9% and 61.9%, respectively. However, the results indicated stability in formal public and private sector employment during the pandemic compared to before the pandemic. Approximately 85% of men and women employed in the formal public sector before the pandemic remained in that labor market position during the second quarter of 2020, although this percentage was slightly larger among women than among men (86.0% vs. 84.3%, respectively). Moreover, around 80% of salaried employees in the formal private sector stayed in that category. However, in the latter case, the stability pattern was slightly lower among women, who also transitioned to inactivity, informal employment, and formal public sector employment.

These results are in line with the changes in the composition of employment statuses described with the cross-sectional data, and they also explain the labor transitions driving these changes in the short-term impact of the pandemic. In particular, the panel data analysis showed that, at the beginning of the pandemic, the rise in inactivity was similar among both men and women (from 14.7% in Q1 2020 to 27.1% in Q2 2020 among men, and from 34.8% to 46.7% among women), and this increase was mainly driven by individuals who were employed in the informal sector, self-employed, or unemployed before the pandemic. Despite these similarities, the results also pointed out incipient gender differences pertaining to job stability: while men were more prone to stay in formal private jobs than women, women were slightly more likely to remain and/or to move into formal public employment than men.

Table 3 also reports the employment transitions from Q4 2020 to Q1 2021 and from Q3 2021 to Q4 2021, which constitute the period of economic recovery and reopening of non-essential activities in the country. The results reinforce the previously described trends. Firstly, the results showed that the Argentine post-pandemic labor market was able to keep men in the workforce at a higher rate than women. Thus, during the first quarter of 2021, men who were previously inactive showed a higher tendency than women to rejoin the labor force. While 30.7% of previously inactive men reentered the labor force in Q1 2021, this percentage was only 19.9% among women, which was approximately 10 percentage points lower than among men. It was in the last quarter of 2021 that the probability of remaining inactive became relatively similar between women (77.4%) and men (75.5%).

Furthermore, the panel data results showed that among those in informal employment, the probability of transitioning to inactivity was larger among women than among men throughout the economic recovery period. Indeed, while 15.3% of women previously employed in the informal sector continued to exit the labor force in Q1 2021, this percentage was only 4.0% among men. This trend persisted during Q4 2021: while 10.7% of women in informal employment in Q3 2021 continued to transition to inactivity in Q4 2021, this percentage was 4.9% among men. Thus, the panel data results during the post-pandemic period suggested that the informal sector proved to be a more stable source of employment for men than for women. A similar gender pattern was seen in self-employment: while 14.2% of self-employed women continued to transition to inactivity in Q4 2021, this percentage was only 5.4% among men.

The results also showed that the formal private sector became increasingly masculinized. On the one hand, the probability of remaining employed in the formal private sector was higher among men than among women. While between Q4 2020 and Q1 2021, the probability of remaining in such position was similar among women (86.3%) and men (88.5%), in Q4 2021, only 75.8% of women in formal private employment in the previous quarter continued in that position, compared to 84.3% of men. The results also showed that during Q1 2021, previously unemployed men were re-employed as formal workers in the private sector to a much greater extent than women (10.1% vs. 1.3%, respectively).

Finally, the panel data results confirmed the feminization of the public sector during the longer-term impact of the pandemic through two complementary processes. On the one hand, while most men and women employed in the formal public sector in Q4 2020 remained in the same position at the beginning of 2021, the probability of remaining in public employment was considerably larger among women (90.0%) than among men (84.8%). On the other hand, in Q4 2021, a higher percentage of women previously employed in the formal private sector transitioned to public employment (5.6%) than men (3.2%). These differences could be seen starting from the second quarter of 2021, as displayed in Table A4 in the Appendix A, when public sector employment began to absorb women from other employment positions to a greater extent than men. Likewise, among those who were informally employed, the probability of transitioning to the formal public sector in Q4 2021 was larger among women (3.9%) than among men (1.7%).

To further reinforce these results, the cross-sectional descriptive data on industrial sectors before, during, and after the COVID-19 pandemic in Argentina (Table A5 in the Appendix A) showed three main trends. First, there were no substantial differences among men in the distribution of industrial sectors over the period analyzed. Indeed, except for the Manufacturing, Trade, and Lodging and Food Services sectors, the percentage of men employed across industries remained relatively stable during and after the pandemic. Second, historically gendered branches of activity, such as Domestic Work, showed the largest declines in employment during and after the pandemic. And third, women relied more on industries with high state participation—e.g., education and social and health services—to ameliorate the effects of the economic crisis. The latter may imply that women who were dismissed from their previous jobs found in the public sector a way to remain in the workforce.

In summary, the panel data results showed that, on the one hand, men reentered the labor market sooner than women. On the other hand, the results pointed out gender differences in the recovery of each employment sector. Indeed, informal jobs proved to be a more stable source of employment among men than among women. Furthermore, the probability of remaining employed in the formal private sector was also larger among men than among women. In contrast, formal public employment was more concentrated among women than among men, with women not only having a higher probability of remaining in that same position but also of transitioning from other labor market positions to public employment.

## 5. Discussion and Conclusions

This study examined the COVID-19 pandemic’s immediate and longer-term impacts on Argentina’s labor market with a focus on gender disparities and the mediating role of the public vs. private sectors. Using household survey data, we assessed men and women’s employment trends before, during, and after the pandemic. The study adds to the literature on gender and the economic consequences of the pandemic in two main ways. First, we interacted gender with informality and public and private sector employment. Doing so allowed us to assess the disparate impacts of the pandemic among men and women employed in informal or formal public and private sector employment. Second, we took advantage of the panel design of household surveys for a more precise focus on employment transitions before, during, and after the pandemic.

Overall, it is important to highlight that the labor market post-pandemic recovery in Argentina was quite remarkable. By the fourth quarter of 2021, men and women had in many ways regained their employment positions with some variation in type of employment representation. Gender differences were clear in the timing of the recovery. The most prominent short-term effect of the pandemic was a dramatic increase in inactivity for both men and women, for whom inactivity increased 11 percentage points in the second quarter of 2020 as compared to before the pandemic. For men though, inactivity declined to below pre-pandemic levels in the first quarter of 2021. It took women 6 months longer, that is, the third quarter of 2021, to regain pre-pandemic inactivity levels.

The panel data focused on employment transitions and illuminated the mechanisms producing gender differences. The results showed that it was the relatively slower recovery of informal private sector employment among women that slowed down their labor market reintegration. Informal private sector employment was a driving force reincorporating men into the labor market; however, the same pattern was not visible among women. Moreover, women depended on the recovery and extension of formal public sector employment to regain their economic position. The public sector has been a driving force expanding female employment in Argentina (Gasparini et al. 2015; Organization for Economic Cooperation and Development (OECD) 2016, 2020). The policies of the Argentine government implemented to mitigate the negative economic effects of the pandemic protected formal and public sector employment. The two forces produced a female labor force by the end of the pandemic that was even more dependent on public sector employment than before the pandemic.

In addition, the analysis of transitions showed that the movement out of informal and self-employment that fueled the expansion of inactivity shortly after the pandemic continued among women for a longer period than among men. Women employed in the informal sector and self-employed continued transitioning into inactivity well into the fourth quarter of 2021, while the pattern had basically stopped among men toward the end of 2020. As a result, while the short-term effect of the pandemic was virtually identical for men and women, if we extend the period of consideration, our results show that the negative impact was stronger among women than men.

There are a number of reasons that may explain these trends. The observed gender differences in labor transitions during and after the COVID-19 pandemic in Argentina may have been influenced by a variety of interconnected factors. It appears that women were less likely to return to their jobs, and this could be attributed to several potential reasons. First, it seems to be the case that the economic sectors in which women were predominantly employed, such as domestic work, recovered at a slower pace than male-dominated industries like construction or manufacturing. This slower recovery could have made it more challenging for women to promptly rejoin the labor force. Occupational sex segregation may also play a significant role in explaining these differences. The concentration of women in lower-paying and less secure jobs has historically been a structural issue in many countries, including Argentina. This occupational segregation could have left women in a more vulnerable position during the economic downturn and hindered their ability to bounce back when economic activity improved. Additionally, it may be the case that women’s traditional role as primary caregivers potentially delayed their return to the labor force. The increased caregiving responsibilities brought about by school closures and the heightened demands of healthcare during and after the pandemic may have disproportionately fallen on women. This caregiving burden could have made it difficult for many women to return to the labor market as they had to juggle their work responsibilities with childcare and other family responsibilities. In conclusion, it is possible that the observed gender differences in labor market reintegration during and after the COVID-19 pandemic in Argentina were influenced by a combination of factors, including the slower recovery of employment sectors predominantly occupied by women, occupational sex segregation, and the potential caregiving responsibilities that women often shoulder. Further research and analysis are necessary to gain a more comprehensive understanding of these dynamics and their impact on gender inequality in the labor market.

Overall, the results illustrate the value of panel data for understanding employment transitions and the effect of sudden economic disruptions, such as the pandemic or economic crises. Given Argentina’s history of recurrent financial crises, the approach can be extended to these other situations. Substantively, the results further reinforce the salience of public sector employment for women’s economic position. Here, differences between employment disruptions resulting from the pandemic and financial crises might be important. Public sector employment was protected during the pandemic. Even more, the health needs of the Argentine population, i.e., healthcare workers primarily, actually contributed to its expansion. In times of financial crises though, that is not the case. The austerity and public sector deficit control political choices that result from financial crises might produce a different environment for public sector employment. In such circumstances, the need for reducing fiscal deficits may actually hinder the possibility of expanding public sector employment. Comparative analyses with a focus on employment transitions and sector employment might shed additional light on how gender interacts with informality and the public sector for understanding the economic consequences of sudden crises.

Lastly, it is worth noting that the case of Argentina may offer valuable insight into the experiences of women in other Latin American countries, where they are often overrepresented in public sector employment. While more research is needed to draw conclusive comparisons, the Argentine case serves as a crucial input for predicting how women might navigate similar crises and the extent to which the state can serve as a short-term solution. Moreover, recent political changes, in particular the election of a candidate who campaigned on a libertarian agenda, leave open the question of public sector employment and the implications for gender inequalities in the labor market. This knowledge can guide policymakers and researchers alike in developing strategies to bolster gender equity and economic resilience when facing future challenges in the region.

## Figures and Tables

**Table 1.** Percentage of Employment Status Before, During and After thePandemic by Gender.

	Before		During			After			
	Fourth Quarter	First Quarter	Second Quarter	Third Quarter	Fourth Quarter	First Quarter	Second Quarter	Third Quarter	Fourth Quarter
Employment Status	2019	2020	2020	2020	2020	2021	2021	2021	2021
**Men**									
Inactive	15.9	15.8	27.2	21.1	17.3	14.4	14.8	15.2	15.0
Informal	17.3	17.4	9.6	12.6	15.8	16.7	15.3	15.5	16.8
Public Formal Employment	11.5	11.5	11.6	11.6	11.5	12.2	12.6	12.7	11.6
Private Formal Employment	26.4	26.4	26.7	25.0	24.7	26.9	27.2	26.8	28.9
Unemployed	5.8	6.2	7.0	5.8	6.9	6.1	5.8	5.3	5.0
Self-employed	23.1	22.7	18.0	23.9	23.9	23.7	24.4	24.5	22.8
Total (unweighted)	16,924	14,967	10,716	12,076	12,791	13,517	13,687	14,273	14,707
**Women**									
Inactive	36.7	36.6	48.0	43.2	39.8	37.2	37.3	35.7	35.6
Informal	14.9	14.4	7.7	9.3	10.7	11.7	11.6	13.8	14.0
Public Formal Employment	12.5	12.8	12.8	13.1	12.6	14.1	15.7	15.4	14.1
Private Formal Employment	16.4	16.0	15.2	14.9	15.0	14.8	15.4	15.1	16.7
Unemployed	5.7	6.8	6.6	6.6	6.3	7.8	6.0	5.9	5.2
Self-employed	13.8	13.6	9.7	12.8	15.5	14.4	14.0	14.1	14.4
Total (unweighted)	18,357	16,325	11,790	13,241	13,908	14,778	14,918	15,576	15,933

*Source:* Based on Encuesta Permanente de Hogares 2019–2021, Argentina (EPH). *Notes:* Percentages obtained with weighted observations.

**Table 2.** Differences in Percentages of Employment Status Before, During and After the Pandemic by Gender (First Quarter 2020 Ref) and Statistical Significance (Two-Sample T Test).

	Before		During						After							
	Fourth Quarter 2019–First Quarter 2020		Second Quarter 2020–First Quarter 2020		Third Quarter 2020–First Quarter 2020		Fourth Quarter 2020–First Quarter 2020		First Quarter 2021–First Quarter 2020		Second Quarter 2021–First Quarter 2020		Third Quarter 2021–First Quarter 2020		Fourth Quarter 2021–First Quarter 2020	
**Employment Status**																
**Men**																
Inactive	0.1		11.3	**	5.3	**	1.5	**	−1.4	**	−1.0	**	−0.6		−0.8	*
Informal	−0.1		−7.8	**	−4.8	**	−1.6	**	−0.7		−2.1	**	−1.9	**	−0.6	
Public Formal Employment	0.0		0.1		0.2		0.0		0.7	*	1.1	**	1.2	**	0.1	
Private Formal Employment	−0.1		0.2		−1.4	**	−1.8	**	0.5		0.8		0.4		2.5	**
Unemployed	−0.3		0.8	**	−0.4		0.7	**	0.0		−0.4		−0.8	**	1.2	**
Self-employed	0.3		−4.7	**	1.1	**	1.2	**	0.9	*	1.6	**	1.7	**	0.0	
**Women**																
Inactive	0.2		11.4	**	6.7	**	3.3	**	0.7		0.7		−0.8		−0.9	*
Informal	0.5		−6.7	**	−5.1	**	−3.7	**	−2.7	**	−2.9	**	−0.6		−0.4	
Public Formal Employment	−0.3		0.0		0.3		−0.2		1.3	**	2.9	**	2.6	**	1.3	**
Private Formal Employment	0.4		−0.7	*	−1.0	**	−1.0	**	−1.1	**	−0.5		−0.8	**	0.8	*
Unemployed	−1.1	**	−0.2		−0.2		−0.4		1.0	**	−0.8	**	−0.8	**	−1.6	**
Self-employed	0.3		−3.8	**	−0.7	*	2.0	**	0.9	**	0.5		0.5		0.9	**

*Source:* Based on Encuesta Permanente de Hogares 2019–2021, Argentina (EPH).

*Notes:* Difference statistically significant at * *p* ≤ 0.10 and ** *p* ≤ 0.05.

**Table 3.** Employment Status Transitions During and After the Pandemic by Gender (%).

Men																							
	Employment Status in Q2 2020							Employment Status in Q1 2021								Employment Status in Q4 2021							
Employment Status in Q1 2020	Inact.	Informal	Public Formal	Private Formal	Unemp	Self-Emp	Total	Employment Status in Q4 2020	Inact.	Informal	Public Formal	Private Formal	Unemp	Self-Emp	Total	Employment Status in Q3 2021	Inact.	Informal	Public Formal	Private Formal	Unemp	Self-Emp	Total
Inactive	81.7	2.4	2.0	0.2	7.5	6.2	14.7	Inactive	69.3	8.1	1.2	2.5	8.7	10.3	16.6	Inactive	75.5	6.1	1.6	2.9	5.5	8.3	15.9
Informal	27.6	33.9	1.2	7.7	14.4	15.1	16.7	Informal	4.0	69.7	0.7	4.6	6.3	14.7	16.6	Informal	4.9	62.1	1.7	10.6	5.7	15.1	14.6
Public Formal Employment	2.9	3.9	84.3	5.6	1.1	2.3	11.8	Public Formal Employment	2.0	2.7	84.8	8.4	0.8	1.4	12.4	Public Formal Employment	1.4	1.8	82.4	8.1	1.9	4.5	13.3
Private Formal Employment	3.2	2.9	4.1	84.8	2.9	2.2	27.7	Private Formal Employment	0.8	4.0	3.8	88.5	0.9	1.9	23.6	Private Formal Employment	2.1	5.4	3.2	84.3	1.6	3.4	26.1
Unemployed	39.8	8.0	3.1	1.0	35.5	12.6	6.0	Unemployed	8.3	28.5	2.4	10.1	39.0	11.7	5.6	Unemployed	14.3	21.2	1.1	4.1	42.5	16.8	4.8
Self-employed	29.8	5.0	1.3	2.7	3.8	57.5	23.1	Self-employed	3.4	12.6	1.0	1.4	2.3	79.2	25.2	Self-employed	5.4	13.8	0.9	4.1	3.8	72.0	25.4
Total	27.1	8.9	12.1	26.1	7.4	18.4		Total	13.9	19.0	12.1	24.0	5.6	25.4		Total	15.5	16.2	12.6	26.3	5.4	24.1	
Women																							
	Employment Status in Q2 2020							Employment Status in Q1 2021								Employment Status in Q4 2021							
Employment Status in Q1 2020	Inact.	Informal	Public Formal	Private Formal	Unemp	Self-Emp	Total	Employment Status in Q4 2020	Inact.	Informal	Public Formal	Private Formal	Unemp	Self-Emp	Total	Employment Status in Q3 2021	Inact.	Informal	Public Formal	Private Formal	Unemp	Self-Emp	Total
Inactive	87.7	1.8	1.0	0.6	4.7	4.1	34.8	Inactive	80.1	4.3	0.8	0.7	7.7	6.4	38.0	Inactive	77.4	8.2	1.7	1.1	4.1	7.6	36.3
Informal	41.5	38.2	1.6	3.0	8.8	6.9	14.3	Informal	15.3	57.8	1.6	8.8	8.6	8.0	11.0	Informal	10.7	59.0	3.9	12.4	3.4	10.7	12.3
Public Formal Employment	3.9	2.4	86.0	6.1	0.7	0.9	13.8	Public Formal Employment	2.1	1.4	90.0	5.2	0.4	0.8	13.6	Public Formal Employment	4.2	4.1	80.5	7.8	0.5	2.9	16.9
Private Formal Employment	4.0	5.3	7.4	79.4	3.2	0.7	16.8	Private Formal Employment	2.1	3.0	7.0	86.3	0.7	0.9	14.2	Private Formal Employment	4.3	8.1	5.6	75.8	1.5	4.8	15.6
Unemployed	61.9	7.2	1.0	3.1	21.5	5.3	6.7	Unemployed	29.2	10.7	0.3	1.3	50.6	8.1	6.3	Unemployed	42.7	15.8	1.3	5.2	22.6	12.4	5.6
Self-employed	35.9	4.9	0.5	2.2	5.2	51.3	13.7	Self-employed	14.2	7.7	0.5	2.1	6.5	69.1	16.9	Self-employed	14.2	9.0	1.6	2.3	3.7	69.2	13.4
Total	46.7	8.4	13.8	15.4	5.6	10.0		Total	37.0	10.6	13.8	14.6	8.3	15.8		Total	35.1	14.3	15.8	15.6	4.0	15.2	

*Source:* Based on Encuesta Permanente de Hogares 2020–2021, Argentina (EPH). *Notes:* Percentages obtained with weighted observations.

## Data Availability

Publicly available datasets were analyzed in this study. This data can be found here: https://www.indec.gob.ar/indec/web/Institucional-Indec-BasesDeDatos (accessed on 1 February 2024).

## Footnotes

1 Table A1 in the Appendix A lists the full Spanish names and acronyms of the programs referred to below in this section.

2 There are different definitions of informality. For instance, the International Monetary Fund argues that the informal economy consists of activities that have market value but are not formally registered (Deléchat and Medina 2020). The Organization for Economic Cooperation and Development (OECD)/International Labour Organization (ILO) (2019, p. 156) go beyond this general definition and state that “employees are considered to have informal jobs if their employment relationship is, in law or in practice, not subject to national labor legislation, income taxation, social protection or entitlement to certain employment benefits (advance notice of dismissal, severance pay, paid annual or sick leave, etc.).” Despite these different definitions, there is an agreement that informal workers are in a more vulnerable situation than workers in the formal sector given that they are not covered by national regulations.

3 The data also suggest another point that is worth mentioning: women found it more difficult than men not only to get out of inactivity but also out of unemployment. We analyze this (and other trends) in Mertehikian and Parrado (2024).

## Appendix A

**Table A1.** Full Names of Programs in English, in Spanish and their Acronyms in Spanish.

Name of the Program in English	Name of the Program in Spanish	Acronym in Spanish
Emergency Assistance Program for Work and Production	Programa de Asistencia de Emergencia al Trabajoy la Produccion	ATP
Emergency Family Income	Ingreso Familiar de Emergencia	IFE
Productive Recovery Program	Programa de Recuperacion Productiva	REPRO
Universal Child Allowance	Asignacion Universal por Hijo	AUH
Universal Pregnancy Allowance	Asignacion Universal por Embarazo	AUE

**Table A2.** Gender Distribution Within Each Employment Status Before, During and After the Pandemic (%).

	Before			During			After					
	First Quarter			Second Quarter			First Quarter			Fourth Quarter		
Employment Status	2020			2020			2021			2021		
	Men	Women	Total (unweighted)	Men	Women	Total (unweighted)	Men	Women	Total (unweighted)	Men	Women	Total (unweighted)
Inactive	28.6	71.4	8336	34.0	66.0	8567	27.0	73.0	7443	28.7	71.3	7888
Informal	52.8	47.2	4952	53.3	46.7	1934	57.7	42.3	3984	53.5	46.5	4706
Public Formal Employment	45.4	54.6	3808	45.2	54.8	2752	45.3	54.7	3728	44.1	55.9	3946
Private Formal Employment	60.5	39.5	6558	61.4	38.6	4654	63.5	36.5	5828	62.3	37.7	6908
Unemployed	45.7	54.3	2024	49.1	51.0	1519	43.0	57.0	1981	47.9	52.1	1549
Self-employed	60.9	39.2	5616	62.8	37.3	3079	61.1	38.9	5333	60.1	39.9	5648

*Source:* Based on Encuesta Permanente de Hogares 2020–2021, Argentina (EPH). *Notes:* Percentages obtained with weighted observations.

**Table A3.** Employment Status Transitions Before and During the Pandemic by Gender (%).

Men																															
	Employment Status in Q1 2020								Employment Status in Q2 2020								Employment Status in Q3 2020								Employment Status in Q4 2020						
Employment Status in Q4 2019	Inact.	Informal	Public Formal	Private Formal	Unemp	Self-Emp	Total	Employment Status in Q1 2020	Inact.	Informal	Public Formal	Private Formal	Unemp	Self-Emp	Total	Employment Status in Q2 2020	Inact.	Informal	Public Formal	Private Formal	Unemp	Self-Emp	Total	Employment Status in Q3 2020	Inact.	Informal	Public Formal	Private Formal	Unemp	Self-Emp	Total
Inactive	71.3	7.5	2.8	1.3	8.0	9.1	16.5	Inactive	81.7	2.4	2.0	0.2	7.5	6.2	14.7	Inactive	60.7	5.9	0.5	0.9	9.3	22.7	26.3	Inactive	64.7	10.1	0.9	2.2	9.8	12.4	21.7
Informal	7.3	58.4	1.0	7.5	9.7	16.1	17.4	Informal	27.6	33.9	1.2	7.7	14.4	15.1	16.7	Informal	13.1	59.3	3.9	9.9	3.1	10.7	10.4	Informal	4.6	66.0	0.4	9.2	5.5	14.4	13.5
Public Formal Employment	2.8	2.0	83.5	8.3	0.5	3.0	11.5	Public Formal Employment	2.9	3.9	84.3	5.6	1.1	2.3	11.8	Public Formal Employment	1.4	5.1	85.7	5.5	0.3	2.0	10.8	Public Formal Employment	1.0	3.3	87.8	5.5	0.1	2.3	12.6
Private Formal Employment	2.0	5.1	2.9	87.5	1.2	1.3	25.6	Private Formal Employment	3.2	2.9	4.1	84.8	2.9	2.2	27.7	Private Formal Employment	1.4	2.2	3.9	88.9	1.2	2.4	25.7	Private Formal Employment	0.5	2.0	4.0	89.8	1.9	1.7	24.5
Unemployed	22.0	19.4	2.1	6.2	37.2	13.1	5.4	Unemployed	39.8	8.0	3.1	1.0	35.5	12.6	6.0	Unemployed	25.6	23.7	0.4	4.4	27.5	18.4	6.3	Unemployed	14.7	25.5	1.5	5.6	45.4	7.4	6.2
Self-employed	5.9	12.6	1.2	2.1	3.8	74.4	23.7	Self-employed	29.8	5.0	1.3	2.7	3.8	57.5	23.1	Self-employed	3.8	7.1	1.4	1.8	1.8	84.1	20.6	Self-employed	4.6	8.3	2.4	1.3	2.4	81.0	21.5
Total	16.4	17.0	11.4	25.7	6.3	23.3		Total	27.1	8.9	12.1	26.1	7.4	18.4		Total	20.2	11.8	11.1	25.3	5.2	26.4		Total	16.8	15.4	12.9	25.0	6.7	23.2	
Women																															
	Employment Status in Q1 2020								Employment Status in Q2 2020								Employment Status in Q3 2020								Employment Status in Q4 2020						
Employment Status in Q4 2019	Inact.	Informal	Public Formal	Private Formal	Unemp	Self-Emp	Total	Employment Status in Q1 2020	Inact.	Informal	Public Formal	Private Formal	Unemp	Self-Emp	Total	Employment Status in Q2 2020	Inact.	Informal	Public Formal	Private Formal	Unemp	Self-Emp	Total	Employment Status in Q3 2020	Inact.	Informal	Public Formal	Private Formal	Unemp	Self-Emp	Total
Inactive	80.0	5.6	0.6	0.8	6.5	6.6	36.9	Inactive	87.7	1.8	1.0	0.6	4.7	4.1	34.8	Inactive	82.9	5.0	0.7	2.2	4.5	4.8	46.3	Inactive	79.1	4.6	0.6	1.0	6.2	8.5	42.9
Informal	15.0	63.4	3.6	4.9	6.4	6.8	14.7	Informal	41.5	38.2	1.6	3.0	8.8	6.9	14.3	Informal	18.8	59.1	2.9	9.1	3.0	7.1	6.9	Informal	11.4	69.2	2.0	4.9	4.7	7.9	8.2
Public Formal Employment	2.1	1.7	87.9	6.3	1.2	1.0	12.0	Public Formal Employment	3.9	2.4	86.0	6.1	0.7	0.9	13.8	Public Formal Employment	1.8	2.8	87.5	7.0	0.3	0.7	12.5	Public Formal Employment	1.0	1.6	93.4	2.6	0.0	1.4	14.0
Private Formal Employment	2.1	7.1	6.2	80.5	2.3	1.9	15.5	Private Formal Employment	4.0	5.3	7.4	79.4	3.2	0.7	16.8	Private Formal Employment	3.7	2.9	3.2	87.8	0.6	1.7	16.5	Private Formal Employment	1.4	3.2	2.6	89.7	1.4	1.7	14.2
Unemployed	37.2	15.7	2.1	1.8	33.3	9.9	6.0	Unemployed	61.9	7.2	1.0	3.1	21.5	5.3	6.7	Unemployed	37.6	10.9	3.9	1.7	33.5	12.5	7.1	Unemployed	28.7	16.6	2.1	0.4	43.6	8.6	6.2
Self-employed	18.5	7.4	1.0	1.1	5.1	66.9	14.9	Self-employed	35.9	4.9	0.5	2.2	5.2	51.3	13.7	Self-employed	19.8	12.1	0.9	1.2	4.3	61.8	10.7	Self-employed	12.7	6.0	2.0	1.1	3.5	74.6	14.6
Total	37.3	14.7	12.5	14.5	6.6	14.4		Total	46.7	8.4	13.8	15.4	5.6	10.0		Total	45.3	9.3	12.4	17.3	5.3	10.6		Total	38.8	10.3	14.3	14.1	6.4	16.2	

*Source:* Based on Encuesta Permanente de Hogares 2019–2020, Argentina (EPH). *Notes:* Percentages obtained with weighted observations.

**Table A4.** Employment Status Transitions After the Pandemic by Gender (%).

Men																															
	Employment Status in Q1 2021								Employment Status in Q2 2021								Employment Status in Q3 2021								Employment Status in Q4 2021						
Employment Status in Q4 2020	Inact.	Informal	Public Formal	Private Formal	Unemp	Self-Emp	Total	Employment Status in Q1 2021	Inact.	Informal	Public Formal	Private Formal	Unemp	Self-Emp	Total	Employment Status in Q2 2021	Inact.	Informal	Public Formal	Private Formal	Unemp	Self-Emp	Total	Employment Status in Q3 2021	Inact.	Informal	Public Formal	Private Formal	Unemp	Self-Emp	Total
Inactive	69.3	8.1	1.2	2.5	8.7	10.3	16.6	Inactive	71.8	9.1	1.2	2.2	6.3	9.4	14.3	Inactive	68.3	9.1	2.0	1.4	8.8	10.5	14.9	Inactive	75.5	6.1	1.6	2.9	5.5	8.3	15.9
Informal	4.0	69.7	0.7	4.6	6.3	14.7	16.6	Informal	7.5	57.5	1.1	9.9	8.9	15.1	16.9	Informal	4.9	62.7	3.0	12.1	4.5	12.8	14.0	Informal	4.9	62.1	1.7	10.6	5.7	15.1	14.6
Public Formal Employment	2.0	2.7	84.8	8.4	0.8	1.4	12.4	Public Formal Employment	1.1	1.0	86.6	9.8	0.4	1.1	13.2	Public Formal Employment	2.0	3.3	84.8	7.8	0.1	2.0	14.3	Public Formal Employment	1.4	1.8	82.4	8.1	1.9	4.5	13.3
Private Formal Employment	0.8	4.0	3.8	88.5	0.9	1.9	23.6	Private Formal Employment	2.0	3.8	2.7	87.9	1.2	2.5	27.2	Private Formal Employment	0.6	3.2	3.2	88.6	1.6	2.8	25.3	Private Formal Employment	2.1	5.4	3.2	84.3	1.6	3.4	26.1
Unemployed	8.3	28.5	2.4	10.1	39.0	11.7	5.6	Unemployed	14.4	18.8	0.9	2.3	42.1	21.6	5.8	Unemployed	22.0	22.6	0.3	5.7	33.9	15.7	6.0	Unemployed	14.3	21.2	1.1	4.1	42.5	16.8	4.8
Self-employed	3.4	12.6	1.0	1.4	2.3	79.2	25.2	Self-employed	5.4	12.3	1.6	4.9	1.9	74.0	22.6	Self-employed	6.4	13.7	2.2	3.6	4.5	69.6	25.6	Self-employed	5.4	13.8	0.9	4.1	3.8	72.0	25.4
Total	13.9	19.0	12.1	24.0	5.6	25.4		Total	14.3	16.1	12.9	28.4	5.6	22.7		Total	14.2	16.3	14.2	26.7	5.5	23.1		Total	15.5	16.2	12.6	26.3	5.4	24.1	
Women																															
	Employment Status in Q1 2021								Employment Status in Q2 2021								Employment Status in Q3 2021								Employment Status in Q4 2021						
Employment Status in Q4 2020	Inact.	Informal	Public Formal	Private Formal	Unemp	Self-Emp	Total	Employment Status in Q1 2021	Inact.	Informal	Public Formal	Private Formal	Unemp	Self-Emp	Total	Employment Status in Q2 2021	Inact.	Informal	Public Formal	Private Formal	Unemp	Self-Emp	Total	Employment Status in Q3 2021	Inact.	Informal	Public Formal	Private Formal	Unemp	Self-Emp	Total
Inactive	80.1	4.3	0.8	0.7	7.7	6.4	38.0	Inactive	78.8	5.3	3.4	1.5	5.3	5.7	36.8	Inactive	78.1	4.5	1.1	0.8	6.5	9.0	34.6	Inactive	77.4	8.2	1.7	1.1	4.1	7.6	36.3
Informal	15.3	57.8	1.6	8.8	8.6	8.0	11.0	Informal	23.5	53.7	1.6	6.7	8.0	6.5	10.8	Informal	12.3	64.8	4.3	6.9	6.0	5.7	11.6	Informal	10.7	59.0	3.9	12.4	3.4	10.7	12.3
Public Formal Employment	2.1	1.4	90.0	5.2	0.4	0.8	13.6	Public Formal Employment	2.6	1.9	83.9	7.9	0.2	3.5	15.7	Public Formal Employment	9.0	2.4	81.7	4.3	0.2	2.5	17.9	Public Formal Employment	4.2	4.1	80.5	7.8	0.5	2.9	16.9
Private Formal Employment	2.1	3.0	7.0	86.3	0.7	0.9	14.2	Private Formal Employment	2.5	3.8	5.6	86.7	0.4	1.0	14.2	Private Formal Employment	3.4	9.6	9.9	74.5	1.0	1.8	14.2	Private Formal Employment	4.3	8.1	5.6	75.8	1.5	4.8	15.6
Unemployed	29.2	10.7	0.3	1.3	50.6	8.1	6.3	Unemployed	44.0	12.4	2.1	2.6	22.4	16.5	8.2	Unemployed	30.8	18.5	1.6	3.6	30.6	14.9	6.6	Unemployed	42.7	15.8	1.3	5.2	22.6	12.4	5.6
Self-employed	14.2	7.7	0.5	2.1	6.5	69.1	16.9	Self-employed	17.3	10.0	1.2	1.3	6.9	63.4	14.4	Self-employed	19.9	9.6	0.9	5.9	2.5	61.2	15.2	Self-employed	14.2	9.0	1.6	2.3	3.7	69.2	13.4
Total	37.0	10.6	13.8	14.6	8.3	15.8		Total	38.3	11.0	15.7	15.3	5.7	14.0		Total	35.6	13.5	17.1	13.5	5.5	14.8		Total	35.1	14.3	15.8	15.6	4.0	15.2	

*Source:* Based on Encuesta Permanente de Hogares 2020–2021, Argentina (EPH). *Notes:* Percentages obtained with weighted observations.

**Table A5.** Percentage of Employees by Industrial Sector Before, During and After the Pandemic by Gender.

Men	Fourth Quarter 2019	First Quarter 2020	Second Quarter 2020	Third Quarter 2020	Fourth Quarter 2020	First Quarter 2021	Second Quarter 2021	Third Quarter 2021	Fourth Quarter 2021
Primary Activities	2.8	2.9	3.9	2.5	2.2	3.1	3.9	3.6	2.8
Manufacturing Industry	13.7	13.5	14.6	15.0	16.0	15.0	13.8	13.4	14.6
Trade	18.9	20.0	19.2	19.0	17.0	17.4	18.2	19.5	20.3
Transportation, Storage and Communication	11.9	11.3	11.7	12.1	10.9	12.0	12.5	11.8	11.4
Lodging and Food Services	4.0	4.4	3.5	3.5	2.8	2.8	3.8	3.4	3.4
Financial and Business Services	10.5	10.7	11.3	10.6	11.2	12.7	11.1	10.8	11.4
Teaching	3.8	3.4	4.9	4.4	3.8	3.5	3.7	3.7	4.3
Social and Health services	3.6	3.2	3.4	4.0	3.8	3.4	3.3	3.4	3.3
Construction	16.0	15.1	11.7	15.1	18.2	14.8	14.7	14.9	14.4
Public Administration and Defense	8.7	9.4	10.6	9.0	8.5	9.4	9.8	10.0	8.6
Other Community, Social and Personal Services	5.4	5.6	4.9	4.6	5.4	5.8	5.1	5.3	5.4
Domestic Work	0.8	0.6	0.4	0.3	0.2	0.1	0.3	0.2	0.2
Total (unweighted)	16,924	14,967	10,716	12,076	12,791	13,517	13,687	14,273	14,707
Women	Fourth Quarter 2019	First Quarter 2020	Second Quarter 2020	Third Quarter 2020	Fourth Quarter 2020	First Quarter 2021	Second Quarter 2021	Third Quarter 2021	Fourth Quarter 2021
Primary Activities	0.6	1.0	0.8	1.0	0.4	0.8	1.1	0.9	0.4
Manufacturing Industry	7.6	8.2	9.1	7.8	10.1	8.2	7.1	6.6	8.4
Trade	16.9	16.5	16.0	16.6	16.9	19.0	17.4	18.0	17.9
Transportation, Storage and Communication	2.5	2.5	1.9	2.0	2.0	2.8	2.7	2.7	2.7
Lodging and Food Services	4.4	4.0	2.2	4.1	3.0	3.2	3.2	3.5	3.8
Financial and Business Services	10.4	10.3	10.5	10.2	10.8	9.6	10.6	10.5	11.3
Teaching	14.3	14.2	17.6	15.9	15.3	14.4	15.7	14.3	15.1
Social and Health services	10.2	10.1	12.5	11.5	11.5	12.1	11.0	11.5	10.4
Construction	1.0	0.4	0.8	0.8	0.5	0.6	0.7	0.8	1.1
Public Administration and Defense	8.6	9.1	10.5	9.6	8.9	10.0	11.0	10.8	9.4
Other Community, Social and Personal Services	7.2	7.2	4.7	6.1	6.3	6.9	6.4	6.4	7.5
Domestic Work	16.3	16.6	13.6	14.4	14.5	12.7	13.2	14.0	12.2
Total (unweighted)	18,357	16,325	11,790	13,241	13,908	14,778	14,918	15,576	15,933

*Source:* Based on Encuesta Permanente de Hogares 2019–2021, Argentina (EPH). *Notes:* Percentages obtained with weighted observations.

## References

[R1] Ameijeiras Analía, Cerrutti Marcela, and Parrado Emilio A.. 2021. Impactos del COVID19 en el mercado de trabajo en la Argentina: Las transiciones laborales y sus determinantes. Paper presented at the XVI Jornadas Argentinas de Estudios de Población. III Congreso Internacional de Población del Cono Sur. Asociación de Estudios de Población de la Argentina, Virtual, Argentina, October 12–15.

[R2] Arnold Jens, Caldera-Sánchez Aida, Paula Garda, Pandiella Alberto González, and Pereira Álvaro S.. 2021. América Latina tras el COVID-19: Cómo impulsar una recuperación tan deseada. Ecoscope. An Economic Lens on Policies for Growth and Wellbeing. Available online: https://oecdecoscope.blog/2021/05/31/america-latina-tras-el-covid-19-como-impulsar-una-recuperacion-tan-deseada/ (accessed on 1 February 2024).

[R3] Arreaza Adriana, López Oswaldo, and Toledo Manuel. 2021. La pandemia del COVID-19 en América Latina: Impactos y perspectivas. Documentos de políticas para el desarrollo No. 1. Serie: Iniciativas para la recuperación en la pospandemia. Caracas: CAF—Banco de Desarrollo de América Latina.

[R4] Arza Camila. 2020. II. Familias, cuidado y desigualdad. In Cuidados y mujeres en tiempos de COVID-19: La experiencia en la Argentina. Documentos de Proyectos (LC/TS.2020/153). Santiago: Economic Commission for Latin America and the Caribbean (ECLAC), pp. 45–65. Available online: https://repositorio.cepal.org/server/api/core/bitstreams/2f102cc4-a758-485e-9f48-799da720ae60/content (accessed on 1 February 2024).

[R5] Batthyány Karina, and Sánchez Agustina Sol. 2020. Profundización de las brechas de desigualdad por razones de género: El impacto de la pandemia en los cuidados, el mercado de trabajo y la violencia en América Latina y el Caribe. Astrolabio Nueva Época. Córdoba: Centro de Investigaciones y Estudios sobre Cultura y Sociedad, Universidad Nacional de Córdoba, vol. 25.

[R6] Bergallo Paola, Mangini Marcelo, Magnelli Mariela, and Bercovich Sabina. 2021. Los impactos del COVID-19 en la autonomía económica de las mujeres en América Latina y el Caribe. COVID-19, Serie de Documentos de Política Pública 25. New York: Programa de las Naciones Unidas para el Desarrollo América Latina y el Caribe.

[R7] Calvo Ernesto, and Murillo M. Victoria. 2012. Argentina: The persistence of peronism. Journal of Democracy 23: 148–61.

[R8] Castagna Alicia Inés, Di Capua Laura, Gutiérrez Silvia Adriana, and Véntola Verónica Andrea. 2021. Dinámica del mercado de trabajo del Aglomerado Gran Rosario en los últimos cinco años. Paper presented at the 25th Conference, Investigaciones en la Facultad de Ciencias Económicas y Estadística, Universidad Nacional de Rosario, Santa Fe, Argentina, April 26–30.

[R9] Center on Budget and Policy Priorities. 2024. Chart Book: Tracking the Recovery from the Pandemic Recession. Available online: https://www.cbpp.org/research/economy/tracking-the-recovery-from-the-pandemic-recession (accessed on 1 February 2024).

[R10] Collins Caitlyn, Ruppanner Leah, Landivar Liana Christin, and Scarborough William J.. 2021. The Gendered Consequences of a Weak Infrastructure of Care: School Reopening Plans and Parents’ Employment during the COVID-19 Pandemic. Gender & Society 35: 180–93.

[R11] Dávila Amparo Armas. 2008. Empleo público en el Ecuador: Una mirada desde el género. Quito: Consejo Nacional de las Mujeres (CONAMU), Fundación Friedrich Ebert-Instituto Latinoamericano de Investigaciones Sociales (FES-ILDIS), Internacional de Servicios Públicos (ISP), and Secretaría Nacional Técnica de Desarrollo de Recursos Humanos y Remuneraciones del Sector Público (SENRES).

[R12] Deléchat Corinne, and Medina Leandro. 2020. What Is the Informal Economy? In Finance & Development. Washington, DC: International Monetary Fund. Available online: https://www.imf.org/en/Publications/fandd/issues/2020/12/what-is-the-informal-economy-basics (accessed on 1 February 2024).

[R13] Diéguez Gonzalo, and Gasparin José. 2016. El rompecabezas del empleo público en Argentina: Quiénes hacen funcionar la maquinaria del Estado? Área de Estado y Gobierno, Programa de Gestión Pública, Documento de Políticas Públicas/Análisis No. 162. Buenos Aires: Centro de Implementación de Políticas Públicas para la Equidad y el Crecimiento (CIPPEC).

[R14] Dunatchik Allison, Gerson Kathleen, Glass Jennifer, Jacobs Jerry A., and Stritzel Haley. 2021. Gender, parenting, and the rise of remote work during the pandemic: Implications for domestic inequality in the United States. Gender & Society 35: 194–205. 10.1177/08912432211001301 PMC9122150 35599685

[R15] Economic Commission for Latin America and the Caribbean (ECLAC), Food and Agriculture Organization of the United Nations (FAO), United Nations Women (UN Women), United Nations Development Program (UNDP), and International Labour Organization (ILO). 2013. Trabajo decente e igualdad de género: Políticas para mejorar el acceso y la calidad del empleo de las mujeres en América Latina y el Caribe. Regional Report. Santiago de Chile: ECLAC, FAO, UN Women, UNDP, ILO.

[R16] Ernst Christoph, and Mourelo Elva López. 2020. La COVID-19 y el mundo del trabajo en Argentina: Impacto y respuestas de política. Technical note. Buenos Aires: Oficina de País de la Organización Internacional del Trabajo para la Argentina.

[R17] Farné Stefano. 2016. Programas de empleo público en América Latina. Serie Macroeconomía del Desarrollo No. 185. Santiago: Economic Commission for Latin America and the Caribbean (ECLAC).

[R18] Farré Lidia, Fawaz Yarine, Libertad González, and Jennifer Graves. 2020. How the COVID-19 Lockdown Affected Gender Inequality in Paid and Unpaid Work in Spain. Discussion paper No. 13434. Bonn: IZA—Institute of Labor Economics.

[R19] Fernández Ana Laura, and González Mariana L.. 2020. Empleo público en Argentina: Características y cambios en su composición y formas de contratación entre 2003 y 2018. Trabajo y Sociedad 35: 545–71.

[R20] Frisancho Verónica, Bustelo Monserrat, and Viollaz Mariana. 2023. What Is the Labor Market Like for Women in Latin America and the Caribbean? Washington, DC: Inter-American Development Bank. Available online: https://publications.iadb.org/en/what-labor-market-women-latin-america-and-caribbean-0 (accessed on 1 February 2024).

[R21] Gasparini Leonardo, Arcidiácono Malena, Carella Laura, Puig Jorge, Gluzmann Pablo, and Brassiolo Pablo. 2015. El empleo público en América Latina. Evidencia de las encuestas de hogares. El Trimestre Económico LXXXII: 749–84.

[R22] Gornick Janet C., and Jacobs Jerry A.. 1998. Gender, the welfare state, and public employment: A comparative study of seven industrialized countries. American Sociological Review 63: 688–710.

[R23] Gutiérrez Diana, Martin Guillermina, and Ñopo Hugo. 2020. El Coronavirus y los retos para el trabajo de las mujeres en América Latina. COVID-19, Serie de Documentos de Política Pública 18. New York: Programa de las Naciones Unidas para el Desarrollo América Latina y el Caribe.

[R24] Hipp Lena, and Bünning Mareike. 2021. Parenthood as a Driver of Increased Gender Inequality during COVID-19? Exploratory Evidence from Germany. European Societies 23: S658–S673.

[R25] INDEC. 2021a. Informe de avance del nivel de actividad. Primer trimestre de 2021. Cuentas nacionales. vol. 5, No. 9. Informes técnicos, vol. 5, No. 113. Instituto Nacional de Estadística y Censos, Ministerio de Economía de la Nación, República Argentina. Available online: https://www.indec.gob.ar/uploads/informesdeprensa/pib_06_21842C1D1A16.pdf (accessed on 1 February 2024).

[R26] INDEC. 2021b. Informe de avance del nivel de actividad. Cuarto trimestre de 2021. Cuentas nacionales. vol. 6, No. 5. Informes técnicos. vol. 6, No. 533. Instituto Nacional de Estadística y Censos, Ministerio de Economía de la Nación, República Argentina. Available online: https://www.indec.gob.ar/uploads/informesdeprensa/pib_03_229F2B413BEF.pdf (accessed on 1 February 2024).

[R27] International Labour Organization (ILO). 2021. COVID-19 and the World of Work, 7th ed. Updated Estimates and Analysis. Available online: https://www.ilo.org/wcmsp5/groups/public/---dgreports/---dcomm/documents/briefingnote/wcms_767028.pdf (accessed on 1 February 2024).

[R28] Manzano Fernando. 2015. El mercado de trabajo femenino en Argentina. Evolución de sus principales indicadores desde el año 1869 al 2010. Paper presented at the XI Jornadas de Sociología, Facultad de Ciencias Sociales, Universidad de Buenos Aires, Buenos Aires, Argentina, July 13–17.

[R29] Manzo Lidia Katia C., and Minello Alessandra. 2020. Mothers, Childcare Duties, and Remote Working under COVID-19 Lockdown in Italy: Cultivating Communities of Care. Dialogues in Human Geography 10: 120–23.

[R30] Marchionni Mariana, Gasparini Leonardo, and Edo María. 2019. Brechas de género en América Latina. Un estado de situación. Caracas: CAF.

[R31] Marshall Adriana. 1990. Introducción: El empleo público ante la crisis fiscal. In El empleo público frente a la crisis. Estudios sobre América Latina. Edited by Marshall Adriana. Ginebra: International Labour Organization, pp. 1–19.

[R32] Matovich Iván, and Bucciarelli María Eugenia. 2021. El regreso a clases 2021: Un análisis de los protocolos provinciales. CIPPEC. Available online: https://www.cippec.org/textual/el-regreso-a-clases-2021-un-analisis-de-los-protocolos-provinciales/ (accessed on 1 February 2024).

[R33] Mera Manuel, Karczmaczyk Matilde, and Petrone Luciana. 2021. El mercado laboral en Argentina: Estructura, impacto del COVID-19 y lecciones para el futuro. Documento de trabajo No. 198. Buenos Aires: Centro de Implementación de Políticas Públicas para la Equidad y el Crecimiento (CIPPEC).

[R34] Mertehikian Yasmin A., and Parrado Emilio A.. 2024. The Impact of the COVID-19 Pandemic on the Labor Market in Argentina. The Intersection of Gender and Socioeconomic Background. Sociology of Development, 1–41. 10.1525/sod.2023.0013 PMC11735040 39823089

[R35] Mertehikian Yasmin A., and Gonalons-Pons Pilar. 2022. Work and Family Disadvantage: Determinants of Gender Gaps in Paid Work during the COVID-19 Pandemic. Socius 8. 10.1177/23780231221117649 PMC9433736 36068884

[R36] Ministerio de Trabajo, Empleo y Seguridad Social. 2017. Las mujeres en el mercado de trabajo. Argentina. Available online: https://www.argentina.gob.ar/sites/default/files/mteyss_genero-y-diversidad-sexual-las-mujeres-en-el-mundo-del-trabajo_2017.pdf (accessed on 1 February 2024).

[R37] Observatorio de la Violencia contra las Mujeres. 2018. III Boletín de Estadísticas de Género. Instituto Nacional de las Mujeres, Ministerio de Desarrollo Social de la Nación Argentina. Available online: https://www.argentina.gob.ar/sites/default/files/3boletinestadisticasdegenero.pdf (accessed on 1 February 2024).

[R38] Organization for Economic Cooperation and Development (OECD). 2016. Panorama de las Administraciones Públicas: América Latina y el Caribe 2017. Paris: OECD Publishing. Available online: https://read.oecd-ilibrary.org/governance/panorama-de-las-administraciones-publicas-america-latina-y-el-caribe-2017_9789264266391-es#page1 (accessed on 1 February 2024).

[R39] Organization for Economic Cooperation and Development (OECD). 2020. Government at a Glance: Latin America and the Caribbean 2020. Paris: OECD Publishing. Available online: https://read.oecd-ilibrary.org/governance/government-at-a-glance-latin-america-and-the-caribbean-2020_13130fbb-en#page1 (accessed on 1 February 2024).

[R40] Organization for Economic Cooperation and Development (OECD)/International Labour Organization (ILO). 2019. Tackling Vulnerability in the Informal Economy. Development Centre Studies. Paris: OECD Publishing. Available online: https://read.oecd-ilibrary.org/development/tackling-vulnerability-in-the-informal-economy_939b7bcd-en#page1 (accessed on 1 February 2024).

[R41] Passerino Leila Martina, and Trupa Noelia Soledad. 2020. Experiencias de cuidados y trabajo: Preocupaciones, malestares y emociones en contexto de pandemia de COVID-19 en Argentina. Revista Feminismos 8: 134–48.

[R42] Reichelt Malte, Makovi Kinga, and Sargsyan Anahit. 2021. The Impact of COVID-19 on Gender Inequality in the Labor Market and Gender-Role Attitudes. European Societies 23: S228–S245.

[R43] Richter Felix. 2021. COVID-19 has caused a huge amount of lost working hours. World Economic Forum. Available online: https://www.weforum.org/agenda/2021/02/covid-employment-global-job-loss/ (accessed on 1 February 2024).

[R44] Rodríguez Laura. 2021. Los retos y oportunidades de la educación secundaria en América Latina y el Caribe durante y después de la pandemia. Santiago: ECLAC. Available online: https://www.cepal.org/es/enfoques/retos-oportunidades-la-educacion-secundaria-america-latina-caribe-durante-despues-la (accessed on 1 February 2024).

[R45] Ruiz Guillermo Ramón, and Cornaglia Emilio. 2023. Impactos del cierre de escuelas sobre el derecho a la educación en la Argentina. Revista Internacional de Teoría e Investigación Educativa 1: 1–9.

[R46] Rulli Mariana. 2020. Perfil de país según igualdad de género. ONU Mujeres Argentina. Available online: https://argentina.un.org/sites/default/files/2020-12/PPIG_Argentina-fn_ESP_2020.pdf (accessed on 1 February 2024).

[R47] Schmidt Manfred. 1993. Gendered Labor Force Participation. In Families of Nations. Patterns of Public Policy in Western Democracies. Edited by Castles Francis G.. Aldershot: Dartmouth, pp. 179–237.

[R48] Schwal Mariano Anderete. 2022. El confinamiento y la vuelta a clases en Argentina: Relatos de docentes sobre la desigualdad en pandemia. Texto Livre 15: e38009.

[R49] Secretaría de Gestión y Empleo Público de la Nación. 2022. Brecha de género en los cargos de gobierno Argentina 2009–2022. Argentina: Jefatura de Gabinete de Ministros. Available online: https://www.argentina.gob.ar/sites/default/files/brecha_de_genero-datos_junio_2022.pdf (accessed on 1 February 2024).

[R50] United Nations Educational, Scientific and Cultural Organization (UNESCO). 2021. Global Monitoring of School Closures Caused by the COVID-19 Pandemic. Available online: https://covid19.uis.unesco.org/data/ (accessed on 1 February 2024).

[R51] United Nations International Children’s Emergency Fund (UNICEF). 2021. COVID-19 and School Closures. One Year of Education Disruption. Available online: https://data.unicef.org/resources/one-year-of-covid-19-and-school-closures/#:~:text=We%20are%20facing%20a%20COVID,will%20pay%20the%20heaviest%20price (accessed on 1 February 2024).

[R52] Wigdor Gabriela Bard, and Bonavitta Paola. 2021. COVID-19, teletrabajo y cuidados: Impacto en la vida de las mujeres profesionales de Argentina. Revista Latinoamericana de Antropología del Trabajo 5: 9–29.

[R53] Zamarro Gema, and Prados María J.. 2021. Gender Differences in Couples’ Division of Childcare, Work and Mental Health during COVID-19. Review of Economics of the Household 19: 11–40. 33488316 10.1007/s11150-020-09534-7 PMC7811157

